# Immunomodulatory and Anti-fibrotic Effects Following the Infusion of Umbilical Cord Mesenchymal Stromal Cells in a Critically Ill Patient With COVID-19 Presenting Lung Fibrosis: A Case Report

**DOI:** 10.3389/fmed.2021.767291

**Published:** 2021-11-17

**Authors:** Kátia Nunes da Silva, Priscila Carvalho Guedes Pinheiro, André Luiz Nunes Gobatto, Rogério da Hora Passos, Bruno Diaz Paredes, Luciana Souza de Aragão França, Carolina Kymie Vasques Nonaka, Beatriz Barreto-Duarte, Mariana Araújo-Pereira, Rafael Tibúrcio, Fernanda Ferreira Cruz, Gabriele Louise Soares Martins, Bruno B. Andrade, Hugo Caire de Castro-Faria-Neto, Patricia Rieken Macêdo Rocco, Bruno Solano de Freitas Souza

**Affiliations:** ^1^Center for Biotechnology and Cell Therapy, São Rafael Hospital, Salvador, Brazil; ^2^D'Or Institute for Research and Education (IDOR), Salvador, Brazil; ^3^Gonçalo Moniz Institute, Oswaldo Cruz Foundation (FIOCRUZ), Salvador, Brazil; ^4^Critical Care Unit, São Rafael Hospital, Salvador, Brazil; ^5^Curso de Medicina, Universidade Salvador (UNIFACS), Laureate International Universities, Salvador, Brazil; ^6^Programa de Pós-Graduação em Clínica Médica, Universidade Federal do Rio de Janeiro, Rio de Janeiro, Brazil; ^7^Multinational Organization Network Sponsoring Translational and Epidemiological Research (MONSTER) Initiative, Salvador, Brazil; ^8^Faculdade de Medicina, Universidade Federal da Bahia, Salvador, Brazil; ^9^Laboratory of Pulmonary Investigation, Carlos Chagas Filho Institute of Biophysics, Federal University of Rio de Janeiro, Rio de Janeiro, Brazil; ^10^COVID-19 Virus Network from Brazilian Council for Scientific and Technological Development, Brasília, Brazil; ^11^COVID-19 Virus Network from Foundation Carlos Chagas Filho Research Support of the State of Rio de Janeiro, Rio de Janeiro, Brazil; ^12^National Institute of Science and Technology for Regenerative Medicine, Rio de Janeiro, Brazil; ^13^Laboratory of Immunopharmacology, Oswaldo Cruz Institute, Oswaldo Cruz Foundation (FIOCRUZ), Rio de Janeiro, Brazil

**Keywords:** COVID-19, ARDS, mesenchymal stromal cells (MSCs), cell therapy, immunomodulation, fibrosis

## Abstract

**Background:** The patients with coronavirus disease 2019 (COVID-19) associated with severe acute respiratory distress syndrome (ARDS) may require prolonged mechanical ventilation which often results in lung fibrosis, thus worsening the prognosis and increasing fatality rates. A mesenchymal stromal cell (MSC) therapy may decrease lung inflammation and accelerate recovery in COVID-19. In this context, some studies have reported the effects of MSC therapy for patients not requiring invasive ventilation or during the first hours of tracheal intubation. However, this is the first case report presenting the reduction of not only lung inflammation but also lung fibrosis in a critically ill long-term mechanically ventilated patient with COVID-19.

**Case Presentation:** This is a case report of a 30-year-old male patient with COVID-19 under invasive mechanical ventilation for 14 days in the intensive care unit (ICU), who presented progressive clinical deterioration associated with lung fibrosis. The symptoms onset was 35 days before MSC therapy. The patient was treated with allogenic human umbilical-cord derived MSCs [5 × 10^7^ (2 doses 2 days interval)]. No serious adverse events were observed during and after MSC administration. After MSC therapy, PaO_2_/FiO_2_ ratio increased, the need for vasoactive drugs reduced, chest CT scan imaging, which initially showed signs of bilateral and peripheral ground-glass, as well as consolidation and fibrosis, improved, and the systemic mediators associated with inflammation decreased. Modulation of the different cell populations in peripheral blood was also observed, such as a reduction in inflammatory monocytes and an increase in the frequency of patrolling monocytes, CD4+ lymphocytes, and type 2 classical dendritic cells (cDC2). The patient was discharged 13 days after the cell therapy.

**Conclusions:** Mesenchymal stromal cell therapy may be a promising option in critically ill patients with COVID-19 presenting both severe lung inflammation and fibrosis. Further clinical trials could better assess the efficacy of MSC therapy in critically ill patients with COVID-19 with lung fibrosis associated with long-term mechanical ventilation.

## Background

SARS-CoV-2 infections present different clinical presentations (1). Severe pneumonia and acute respiratory failure may occur, often requiring long-term hospitalization in the intensive care units (ICUs) and prolonged ventilatory assistance (2). Patients with severe/critical coronavirus disease 2019 (COVID-19) usually present a hyperinflammatory and hypercoagulable state, which may also compromise multiple organs (3). The prognosis of patients with COVID-19 has been associated with age, comorbidities, and duration of mechanical ventilation (4, 5). The latter may result in lung fibrosis, thus, impairing gas exchange and increasing the fatality rates.

To date, few pharmacological measures, such as dexamethasone, have been shown to decrease mortality in mechanically ventilated patients with COVID-19 (6, 7); however, this has not been observed in the critically ill patients with COVID-19 with lung fibrosis associated with long-term mechanical ventilation. Despite significant therapeutic advances with increased knowledge and definition of standard protocols, critical COVID-19 remains a life-threatening disease and novel therapeutic strategies are urgently needed.

The mesenchymal stromal cells (MSCs) have been evaluated in compassionate use or clinical trials to treat COVID-19 pneumonia (8–19). The rationale of this therapy is to direct the immunomodulatory properties of the MSCs to control the hyperinflammatory state and improve respiratory function (20). Most of the protocols, however, included patients with the early-stage disease or shortly after tracheal intubation. Little attention, however, has been given to late-stage critical cases of acute respiratory distress syndrome (ARDS) associated with COVID-19, in which extensive lung damage and fibrosis have already occurred, thus worsening the prognosis and increasing the fatality rates.

In this study, we report a case of a 30-year-old critically ill long-term mechanically ventilated patient with COVID-19 with severe lung inflammation and fibrosis treated with umbilical cord-derived MSCs (UC-MSCs).

## Case Presentation

A 30-year-old male patient with no known comorbidities presented on Jun 6 with myalgia, headache, shortness of breath with moderate efforts at illness day 3, SpO_2_ > 95%, and a positive test for SARS-CoV-2 by nasopharyngeal reverse transcription (RT)-PCR. At this point, the CT scan showed parenchymal ground-glass opacities in up to 25% of the lung parenchyma. The patient was medicated and discharged, returning at illness day 6 with worsened dyspnea, SpO_2_ = 88% at ambient air, >50% altered lung parenchyma on CT, being classified as severe (21). He was admitted to the ICU at São Rafael Hospital, Salvador, Brazil, receiving oxygen therapy, bronchodilators, anticoagulant, methylprednisolone (120 mg/day), and antibiotics (ceftriaxone + azithromycin). The patient was treated with high-flow nasal oxygen, a non-rebreathing mask, and required pronation to sustain SpO_2_, but did not respond, progressing with desaturation, respiratory acidosis, and septic shock, requiring orotracheal intubation on illness day 15, being clinically diagnosed with critical COVID-19. Laboratory testing was consistent with cytokine storm, with reduced lymphocyte counts, increased C-reactive protein (CRP), D-dimer, lactate dehydrogenase (LDH), fibrinogen, and ferritin, along with PaO_2_/FiO_2_ < 200. The patient required vasoactive drugs to keep mean arterial pressure above 65 mmHg. On the following days, serial SARS-CoV-2 reverse transcription PCR (RT-PCR) results were persistently positive, and secondary infections with *Stenotrophomonas* and *Klebsiella pneumoniae* were detected in the tracheal aspirate, treated with antibiotics. Tracheostomy was performed on Jun 29. The patient presented clinical deterioration on illness day 30 and CT imaging demonstrated radiological worsening with an ARDS pattern, lesions affecting >75% of the lung parenchyma, and foci of interstitial fibrosis. A cell therapy protocol with UC-MSCs was then applied on a compassionate use basis, following informed consent given by the family of the patient.

Mesenchymal stromal cells were obtained from the umbilical cord tissue at a cGMP facility at the Center for Biotechnology and Cell Therapy, São Rafael Hospital, Salvador, Brazil and cryopreserved at passage 3 in 50 ml of a cryopreservation solution containing Plasmalyte, 3% human albumin, and 5% dimethyl sulfoxide (DMSO) and stored in cryobags at <-135°C. The identity of MSC was assessed by flow cytometry (Stemflow Human MSC Analysis kit, BD Biosciences, NJ, USA) and *in vitro* trilineage differentiation assays (StemPro Osteogenesis and Adipogenesis kits, Thermo Fisher Scientific, MA, USA) (Supplementary Figures 1A–E). Genetic stability was evaluated by G-band karyotype, as previously described (22) (Supplementary Figure 1F). Sterility was evaluated by culture for anaerobic, aerobic bacteria and fungi, endotoxin levels, and mycoplasma test. The potency was evaluated by measuring IDO1 mRNA expression by quantitative reverse transcription PCR (RT-qPCR) after stimulation with IFNγ (Supplementary Figure 2A), as described previously (23). Finally, the product hemocompatibility was tested by evaluating tissue factor (CD142) expression by flow cytometry and by performing thromboelastography (TEG) studies in the citrate blood samples obtained from three different donors, as previously described (Supplementary Figures 2B–D) (24). Finally, the cell viability was checked before cryopreservation, 48 h after, and at the time of infusion, by flow cytometry with 7AAD (BD Biosciences) (Supplementary Figure 2E).

Approximately 30–60 min before the infusions, the patient received 50 mg diphenhydramine to prevent the infusion-related allergy. The cells were thawed in a 37°C water bath and immediately taken to the patient bedside for intravenous infusion, *via* gravity, over 30–40 min. The patient was followed up by daily clinical evaluations and laboratory testing. The radiological evaluation was performed by serial chest X-rays and CT scans. Additionally, the blood samples were collected on day 1 (pre-infusion), day 3, and day 7 following treatment initiation with MSCs for the evaluation of cytokines and chemokines by Luminex and immune cell populations by flow cytometry (days 1, 3, 7, and 14 following treatment initiation; antibody information in Supplementary Table 1; gating strategy in Supplementary Figures 3, 4). To evaluate the overall profile of biomarkers, an unsupervised hierarchical cluster with the Luminex and flow cytometry assay values was performed using Ward's method. In this analysis, the dendrograms represent the Euclidean distance (inferring degree of similarity). The values were normalized using the Z-score method. To calculate a fold-change, day 1 was used as a reference.

Umbilical cord-derived-mesenchymal stromal cells are characterized according to their ability to adhere to plastic, and high expression (>95%) of CD90, CD105, CD44, and CD73, and low expression (<2%) of CD45, CD34, and CD117 (Supplementary Figure 1E), and also by their *in vitro* adipogenic, osteogenic, and chondrogenic differentiation, with a normal karyotype (Supplementary Figure 1F) (25).

**Figure 1:**
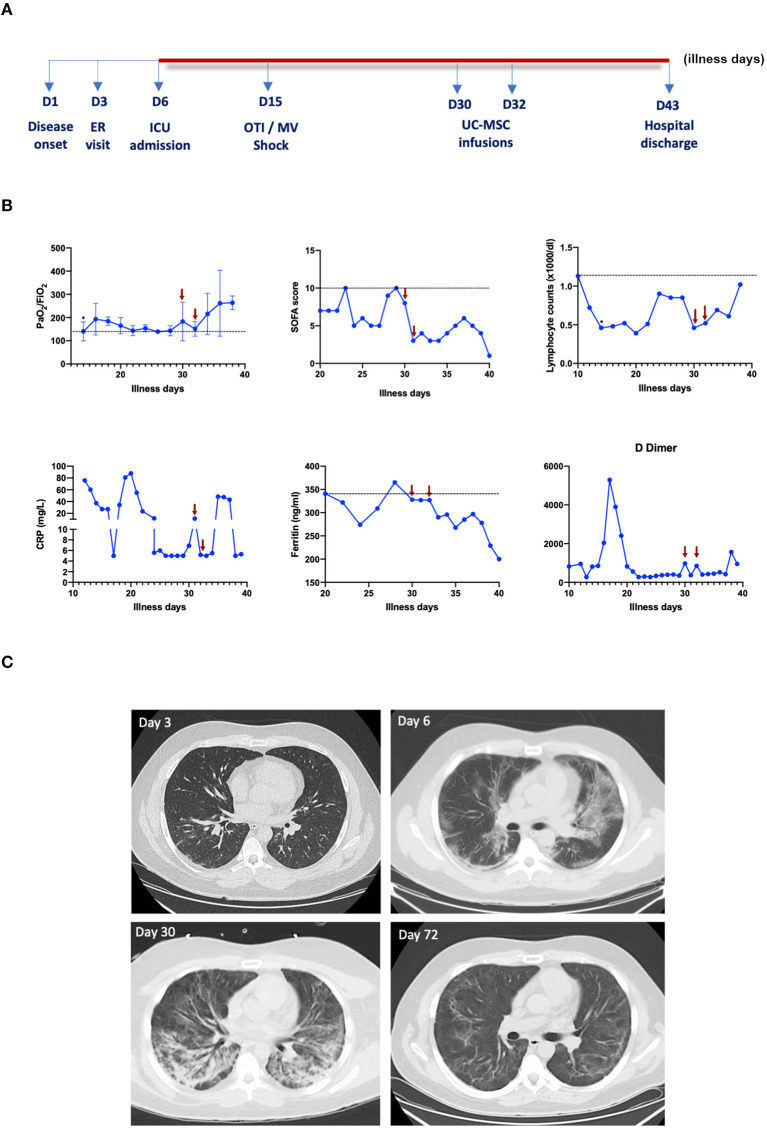
Evolution of clinical, laboratory, and radiological parameters. **(A)** Timeline of the duration of patient stays in the intensive care unit (ICU), shown as illness days. OTI, orotracheal intubation; MV, mechanical ventilation; UC-MSC, umbilical cord-derived mesenchymal stromal cells. Arrows represent the time of MSC infusions; respective samples were collected prior to MSC infusion. *Day of orotracheal intubation. **(B)** Data are represented as single values or mean ± SD of the values of different measurements performed each day. **(C)** The representative images of chest CT scans were performed at different time points.

Two intravenous administrations of UC-MSCs (50 million cells/infusion) were performed at illness days 30 and 32. The infusions were well-tolerated, and no adverse events were observed. The patient showed a rapid improvement in oxygenation, requiring progressively lower levels of vasopressors until hemodynamic stability without vasopressors was achieved 9 days post the UC-MSC infusion. The patient was discharged 13 days after UC-MSC infusion. The variations in PaO_2_/FiO_2_, Sequential Organ Failure Assessment (SOFA) score, lymphocyte counts, CRP, ferritin, and D-dimer, along with the dynamic changes seen in chest CT are shown (Figure 1). The control CT scan showed significant absorption of bilateral pulmonary infiltrates, maintaining only retractable opacities.

**Figure 2:**
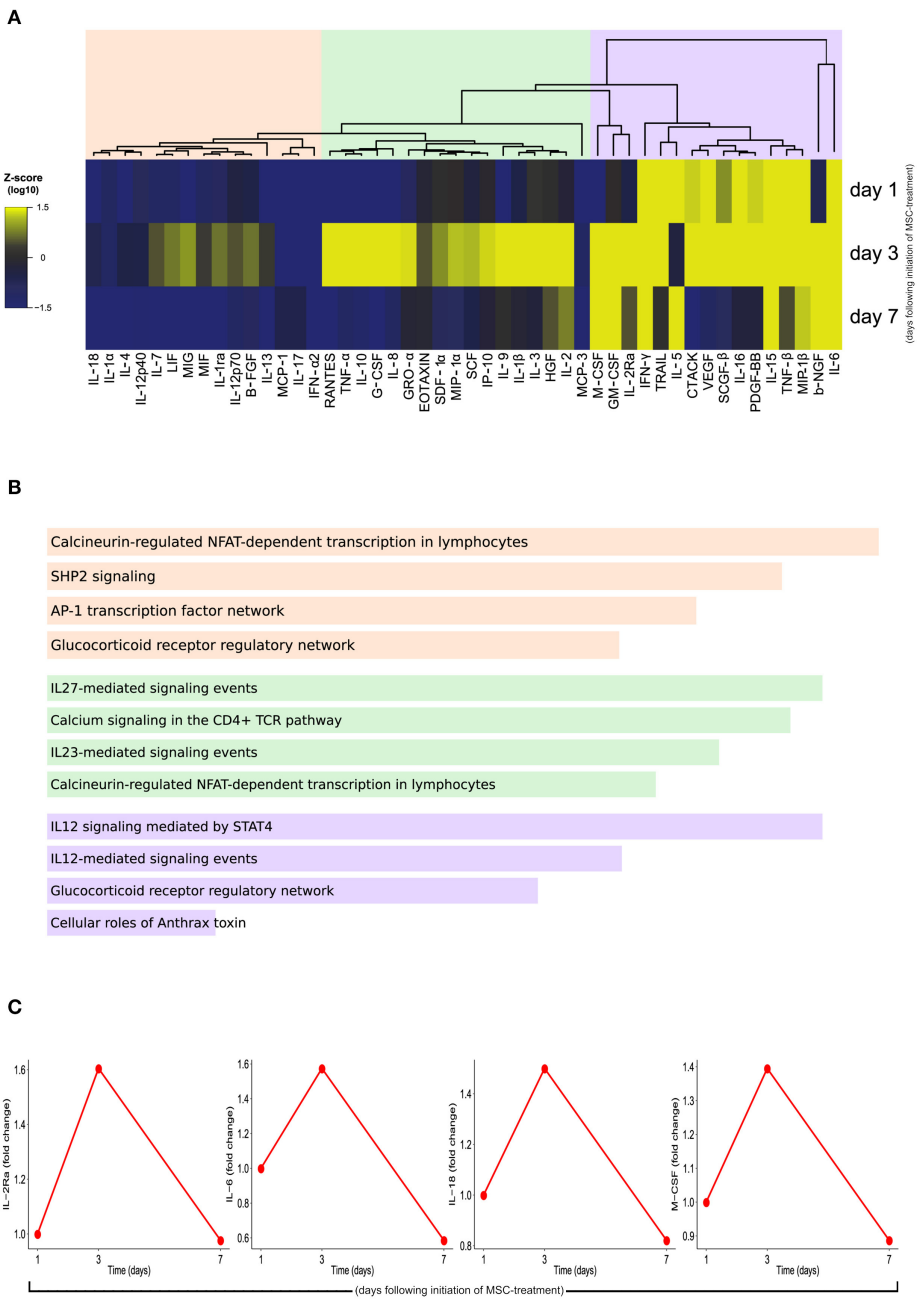
Expression profile of cytokines, chemokines, and growth factors by Luminex assay. **(A)** A heatmap was designed to depict the overall profile of biomarkers among times, according to log_10_ of Mean Fluorescent Intensity (MFI). A one-way hierarchical cluster analysis (Ward's method) was performed. The dendrograms represent Euclidean distance. **(B)** Enrichment analysis for each cluster at NCI Nature database. The top four pathways are shown, ranked by the *p*-value. **(C)** Interlukin (IL)-2Ra, IL-6, IL-18, and macrophage colony-stimulating factor (M-CSF) showed a higher fold-change (±0.5) of MFI over time. The samples were collected on the day of the first MSC infusion (D1, pre-infusion), the second MSC infusion (D3, pre-infusion), and the 7th day following cell therapy (D7).

To evaluate the possible mechanisms of action of MSCs in the immune cells and soluble mediators, we performed the Luminex and flow cytometry analyses follow cell therapy initiation. An unsupervised hierarchical analysis was performed with Luminex data, and three clusters of plasma biomarkers were established. On the first and second clusters, a slight increase in the plasma cytokine levels was observed on day 3, compared with day 1. On day 7, the levels of most cytokines approached the first measured value (Figures 2A,B). On the third cluster, the increase was more accentuated and was maintained at day 7 (Figure 2A). For each cluster, we performed enrichment analysis on the NCI Nature database. The first was enriched mainly to the regulation of transcription and signaling process in the lymphocytes. The second was enriched with IL-27, calcium, and IL-23 signaling. While the third was enriched to IL-12 signaling events (Figure 2B). The biomarkers that showed the greatest discrepancies (±0.4-fold change) at day 3 were IL-2RA, IL-18, IL-6, and M-CSF (Figure 2C).

**Figure 3:**
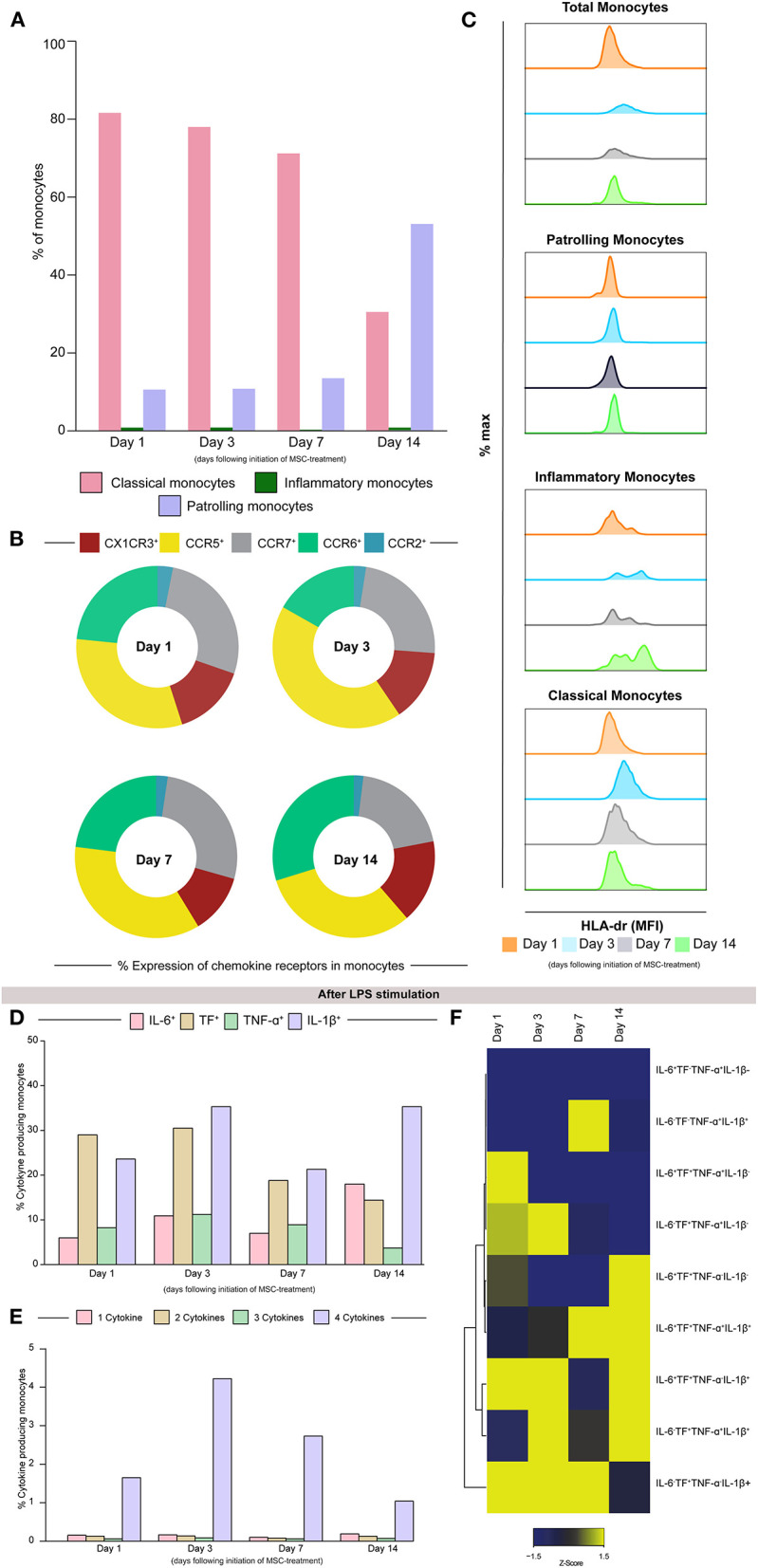
The classical monocytes (CD14^++^CD16^−^) are more prevalent up to 7 days after treatment initiation. **(A)** The frequency of classical (CD14^++^CD16^−^), inflammatory (CD14^++^CD16^+^), and patrolling (CD14^+^CD16^+^) monocytes (pink, green, and purple, respectively), exhibits the same overall profile until day 7. On day 14, there is an increase in the patrolling monocytes frequency, that exceeds 50% of all the monocyte populations. **(B)** Observing the frequencies, we notice that CCR5 and CCR7 are the most prevalent markers over time. **(C)** Median fluorescence intensity of HLD-DR among the monocytes subpopulation over period post-treatment. The assessment of monocyte polyfunctionality following overnight lipopolysaccharide (LPS) stimulation. **(D)** The frequency of IL-6, TF, TNF-a, and IL-1B expression is shown. **(E)** The frequencies of cytokine producer subpopulations are shown. **(F)** A heatmap was designed to depict the overall profile of cytokine producer subpopulation of monocytes overtimes. A one-way hierarchical cluster analysis (Ward's method) was performed. The dendrograms represent Euclidean distance. The samples were collected on the day of the first MSC infusion (D1, pre-infusion), second MSC infusion (D3, pre-infusion), and the 7th and 14th days following cell therapy (D7 and D14).

The flow cytometry analysis demonstrated that up to 7 days following treatment initiation, classical monocytes (CD14^++^CD16^−^) were enriched in the peripheral blood, whereas on day 14, the patrolling monocytes (CD14^+^CD16^++^) were the most prevailing monocyte subpopulation (Figure 3A). The substantial alterations of chemokine receptors expression over time post-treatment, with an increase on CCR5^+^ receptors and decrease of CCR7^+^ (Figure 3B). Additionally, the degree of monocyte activation was substantially altered following the treatment in the inflammatory monocyte subpopulation, where it is possible to observe a decrease in these activated monocytes on day 3 and day 7, with a subsequent increase on day 14, where the profile is similar to the baseline (Figure 3C). Regarding monocyte polyfunctionality in response to a toll-like receptor-4 (TLR-4) agonist [lipopolysaccharide (LPS), 1 μg/ml], there was a peak of multiple cytokine producer monocytes on day 3, suggesting higher polyfunctional activity in this period (Figures 3D–F).

**Figure 4:**
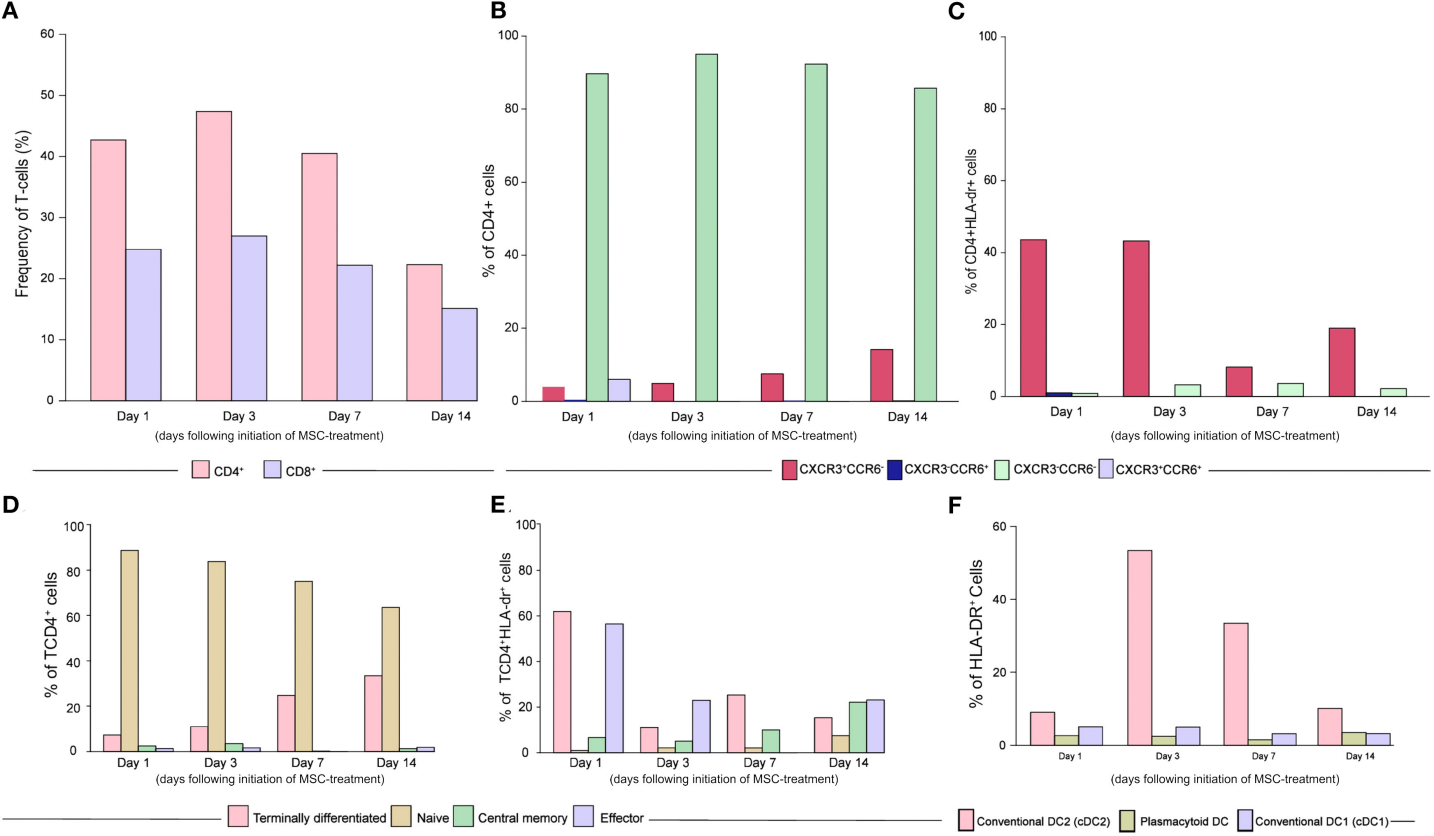
Dynamics of CD4^+^ T helper subsets and dendritic cells turnover following treatment initiation. **(A)** Frequencies of CD4^+^ and CD8^+^ T Lymphocytes over time. **(B)** The differential expression of chemokine receptors CCR6 and CXCR3 in CD4 T cells are shown, further indicating the frequencies of Th1 (CXCR3^+^CCR6^−^), Th1 Star (CXCR3^+^CCR6^+^), Th2 (CXCR3^−^CCR6^−^), and Th17 (CXCR3^−^CCR6^+^) subpopulations. **(C)** The differential expression of chemokine receptors CCR6 and CXCR3 in activated CD4 T cells are shown, further indicating the frequencies of Th1 (CXCR3^+^CCR6^−^), Th1 Star (CXCR3^+^CCR6^+^), Th2 (CXCR3^−^CCR6^−^), and Th17 (CXCR3^−^CCR6^+^) subpopulations. **(D)** The frequencies of naïve (CD45^+^CD27^+^), central memory (CD45^−^CD27^+^), effector (CD45^−^CD27^−^) and terminally differentiated cells (CD45^+^CD27^−^) are shown, without activation. **(E)** The frequencies of naïve (CD45^+^CD27^+^), central memory (CD45^−^CD27^+^), effector (CD45^−^CD27^−^), and terminally differentiated cells (CD45^−^CD27^+^) are shown in activated (HLA^−^DR^+^) cells. **(F)** Conventional dendritic cells subtype 2 (cDC2) frequencies increased on day 3. The frequency of conventional DC2 (HLA^−^dr^+^CD11c^+^CD141^−^), plasmacytoid DCs (HLA^−^dr^+^CD11c^−^CD123^+^), and conventional DC1 (HLA^−^dr^+^CD11c^+^CD141^+^) (pink, brown and light purple, respectively) are shown. Samples were collected on the day of the 1st MSC infusion (D1, pre-infusion), 2nd MSC infusion (D3, pre-infusion) and on the 7th and 14th days following cell therapy (D7 and D14).

The frequencies of CD4^+^ and CD8^+^ T cells in peripheral blood presented similar changes over time following the MSC treatment initiation. In both cases, there was observed a slight increase at day 3, with progressive reduction at day 7 and day 14 (Figure 4A). The evaluation of differential chemokine receptor expression of CCR6 and CXCR3 on circulating CD4^+^ lymphocytes revealed higher frequencies of CXCR3^−^CCR6^−^ (Th2) subpopulation following treatment (Figure 4B). In activated cells, the profile changes and CXCR3^+^CCR6^−^ are more frequent (Figure 4C). Interestingly, the patient exhibited a reduction in naïve CD4^+^ T-cells frequencies over time along with the increased frequencies of terminally differentiated CD4^+^ T cells (Figure 4D). The activated TCD4^+^ cells were more terminally differentiated and effector on the first day, comparing with the other days (Figure 4E). Of note, the conventional dendritic cells 2 (cDC2) frequency peaked on day 3 after treatment, returning to the basal levels afterward (Figure 4F).

## Discussion and Conclusions

The MSC-based therapy protocols to treat COVID-19 have been directed mainly to patients with moderate and severe clinical presentations (8–19). Few studies included critically ill patients with COVID-19 under invasive mechanical ventilation (26–28). Although preliminary, the published data suggest that the critically ill patients who presented benefits with the MSC treatment were successfully extubated after receiving MSCs shortly following intubation (28). The results of the first published randomized controlled trials also support enhanced time to recovery and improved survival following the treatment with MSCs in the patients with COVID-19 ARDS of different severity levels, supporting the trends for improved radiological recovery (29, 30). In this study, we report the case of a patient successfully treated with MSCs 14 days after tracheal intubation and invasive mechanical ventilation, in which time association between MSC infusion and amelioration of clinical, oxygenation, and laboratory parameters were observed. Importantly, intravenous administration of UC-MSCs was not associated with serious adverse events. This is particularly important in the context of severe/critical COVID-19, due to a thromboinflammatory state (31).

The initial anti-SARS-CoV-2 response starts with the activation of innate immune cells, which function as antigen presenters and produce type I interferons (32). As the infection progresses and tissue injury increases, an exacerbated inflammatory response, with the high levels of pro-inflammatory mediators is seen (33). Prolonged exposure to a cytokine storm scenario as an expression of the dysregulated immune response, leads to macrophage activation syndrome, induced by interleukin-1β (IL1β) (34), defects in the antigen presentation induced by IL-6, decreased HLA-DR expression in monocytes, CD4^+^T cell depletion, the rapid spread of the virus, and secondary organ dysfunction (35). Poor innate immune response in severe COVID-19 was recently characterized as immune paralysis, resembling some characteristics of bacterial sepsis (36). Our results demonstrate that, after MSC therapy, the monocytes increased HLA-DR expression and showed an increased ability to respond to TLR4 ligand stimulation. In addition, we observed a marked increase in the frequency of cDC2, accompanied by a transient increase in the serum levels of different cytokines that are involved in antigen presentation, antiviral response, and differentiation of effector CD4^+^ T cells (37). Interestingly, one of the upregulated pathways found was IL-27 signaling, and dendritic cell-derived IL-27 has been associated with the induction of Treg in lung parenchyma and resolution of immunopathology upon infection with respiratory viruses (38).

After day 3, we observed a reduction in proinflammatory cytokines, and the subset of naïve CD4^+^ T cells, along with the increased frequencies of the terminally differentiated subset with Th2 markers. Finally, after day 7, we observed an increased frequency of patrolling monocytes, a population involved in the resolution of inflammation and healing (39), which migrates to the lungs, differentiate into CD11c^+^, resident lung macrophage, and act in a specialized way as effector cells (40). In addition, the patrolling monocytes respond strongly to viruses *via* the TLR7/8-MEK pathway, producing cytokines, such as TNFα and IL1β, as well as CCL5 and CXCL10 chemokines (41, 42).

In summary, the results of this case report support a potential role for MSC-based therapies not only in the early stages of COVID-19, as has been extensively explored, but also in the advanced stages of critical disease facing clinical deterioration. Administration of UC-MSCs at day 30 of illness, and 14 days after orotracheal intubation, was still safe and associated with a significant change in the clinical course. Further clinical studies with proper design and sample size are required to confirm the efficacy of MSC-based therapies in the advanced stages of severe/critical COVID-19.

## Data Availability Statement

The raw data supporting the conclusions of this article will be made available by the authors, without undue reservation.

## Ethics Statement

The studies involving human participants were reviewed and approved by the Ethics Committee of CEP Hospital São Rafael. The patients/participants provided their written informed consent to participate in this study. Written informed consent for publication of their clinical details and/or clinical images was obtained from the patient or the parent/guardian/relative of the patient.

## Author Contributions

BS and PR: conception and design, provision of study material, data analysis and interpretation, manuscript writing, financial support, and final approval of the manuscript. AG and RP: performed the cell infusions, clinical evaluation, data collection, and analysis. GM and PP: data collection and analysis. KS, BP, LF, and CN: cell manufacturing, characterization, and product quality control. FC and HC-F-N: luminex experiments and cytokine analysis. BA, BB-D, MA-P, and RT: flow cytometry evaluation, data analysis, and interpretation. All authors contributed to the article and approved the submitted version.

## Funding

This study was supported by grants from the Serrapilheira Foundation, D'Or Institute for Research and Education (IDOR), CNPq, and CAPES.

## Conflict of Interest

The authors declare that the research was conducted in the absence of any commercial or financial relationships that could be construed as a potential conflict of interest.

## Publisher's Note

All claims expressed in this article are solely those of the authors and do not necessarily represent those of their affiliated organizations, or those of the publisher, the editors and the reviewers. Any product that may be evaluated in this article, or claim that may be made by its manufacturer, is not guaranteed or endorsed by the publisher.

## Supplementary Material

Supplementary Figure 1 Characterization of UC-MSCs. Phase contrast microscopy imaging of UC-MSCs cultured with growth media **(A)**, or exposed to **(B)** adipogenic, **(C)** osteogenic or **(D)** chondrogenic differentiation media. Differentiation was confirmed by Oil red **(B)**, Alizarin red **(C)** or Alcian Blue **(D)** staining. **(E)** Immunophenotype evaluated by flow cytometry analysis for MSC markers CD90, CD44, CD105 and CD73 and a cocktail of negative markers (CD45, CD34 and CD117). Light histograms represent staining with isotype controls. **(D)** G-band karyotype representative image of normal (46, XX) karyotype.

Supplementary Figure 2 Evaluation of potency, hemocompatibility and cell viability. **(A)** MSCs were evaluated for IFN-γ-induced IDO1 mRNA expression. **(B)** Tissue factor (CD142) expression was evaluated by flow cytometry. Light histogram represents staining with isotype control. **(C,D)** Results of thromboelastography analysis using blood/MSC mixture. The analysis was performed with blood donated from three independent healthy donors. Data are represented as mean SD. INTEM: contact-activated assay to evaluate clot formation via the intrinsic pathway; EXTEM: a tissue-factor activated assay to evaluate clot formation via the Extrinsic pathway. CT: clotting time; CFT: clot formation time; MCF: maximum clot firmness; **P* < 0.05. **(E)** Results of cell viability analysis performed before cryopreservation, 48 h after cryopreservation and at the moment of cell infusion, evaluated by 7AAD flow cytometry assay.

Supplementary Figure 3 Gating strategy displaying the analysis of human monocytes. Peripheral Blood mononuclear cells (PBMCs) were stained using the panel indicated in the method section for quantitative analysis of chemokine receptor expression in monocytes. Samples were gated on monocytes based on SSC-A (complexity) and FSC-A (size) **(A)**, single events **(B)** HLA-DR+ and DUMP - cells **(C)**, monocyte subtype based on CD14 and CD16 expression **(D)**, CX1CR3 **(E)**, CCR2 **(F)**, CCR7 **(G)**, CCR6 **(H)**, and CCR5 **(I)** expression.

Supplementary Figure 4 Gating strategy display the analysis of T lymphocytes in regard to memory phenotype and function. Peripheral Blood mononuclear cells (PBMCs) were stained using the panel indicated in the method section for quantitative analysis of memory and function markers in both CD4^+^ and CD8^+^ T cells. Samples were gated on monocytes based on SSC-A (complexity) and FSC-A (size) **(A)**, single events **(B)**, CD3^+^CD45^+^ cells (T lymphocytes) **(C)**, CD4 and CD8 T cells **(D)**, CXCR3 and CCR6 (T helper subtype) **(E)**, and CD45 and CD27 (memory markers) **(F)**.

Supplementary Table 1 List of antibodies used for the flow cytometry analyses.

## Abbreviations

RT-PCR: reverse transcription PCR
cGMP: current good manufacturing practices.
