# Supplementary figures and images for: Immunomodulatory and Anti-fibrotic Effects Following the Infusion of Umbilical Cord Mesenchymal Stromal Cells in a Critically Ill Patient With COVID-19 Presenting Lung Fibrosis: A Case Report
Source: Front Med (Lausanne). 2021 Nov 17;8:767291. doi: 10.3389/fmed.2021.767291 (PMC8635722; doi:10.3389/fmed.2021.767291)

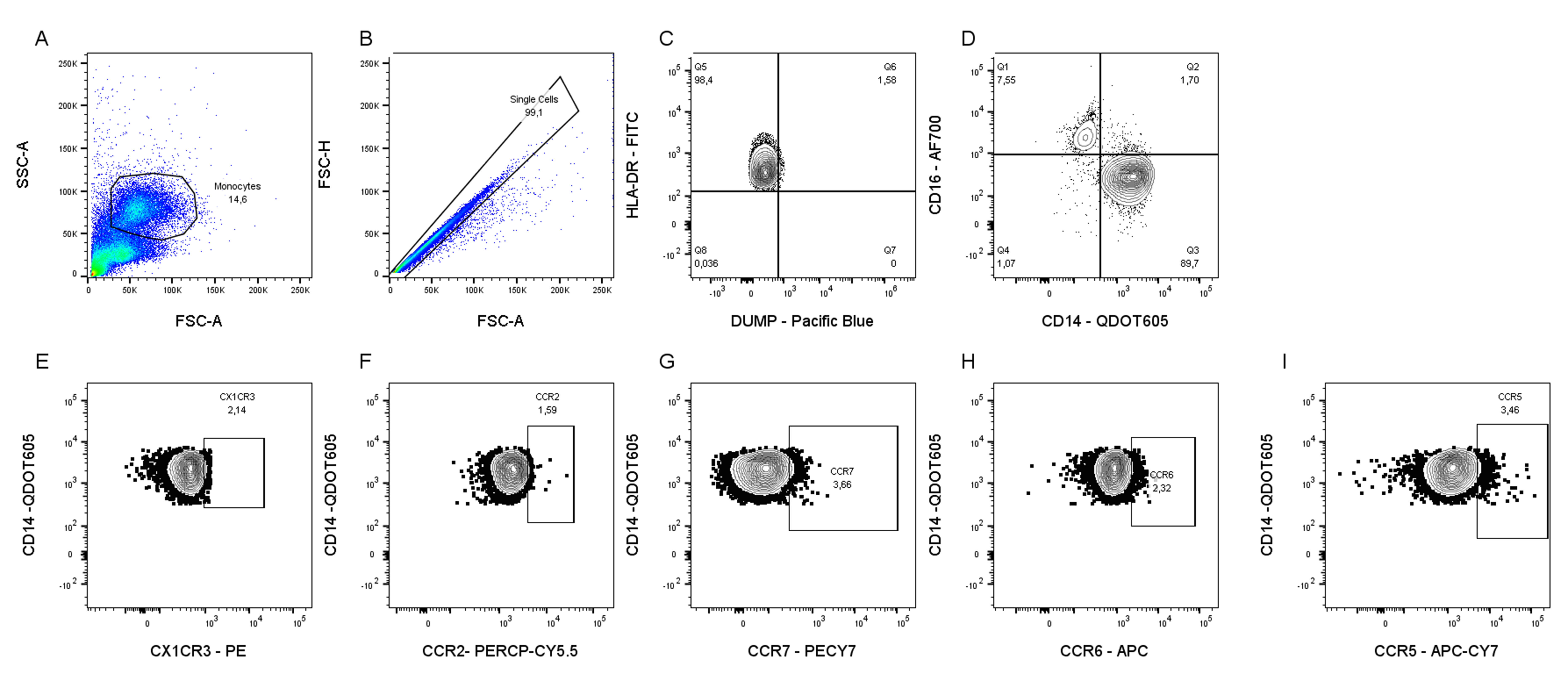

Supplement: Supplementary Figure 3 — Gating strategy displaying the analysis of human monocytes. Peripheral Blood mononuclear cells (PBMCs) were stained using the panel indicated in the method section for quantitative analysis of chemokine receptor expression in monocytes. Samples were gated on monocytes based on SSC-A (complexity) and FSC-A (size) (A), single events (B) HLA-DR+ and DUMP - cells (C), monocyte subtype based on CD14 and CD16 expression (D), CX1CR3 (E), CCR2 (F), CCR7 (G), CCR6 (H), and CCR5 (I) expression. [file Image_3.tiff]

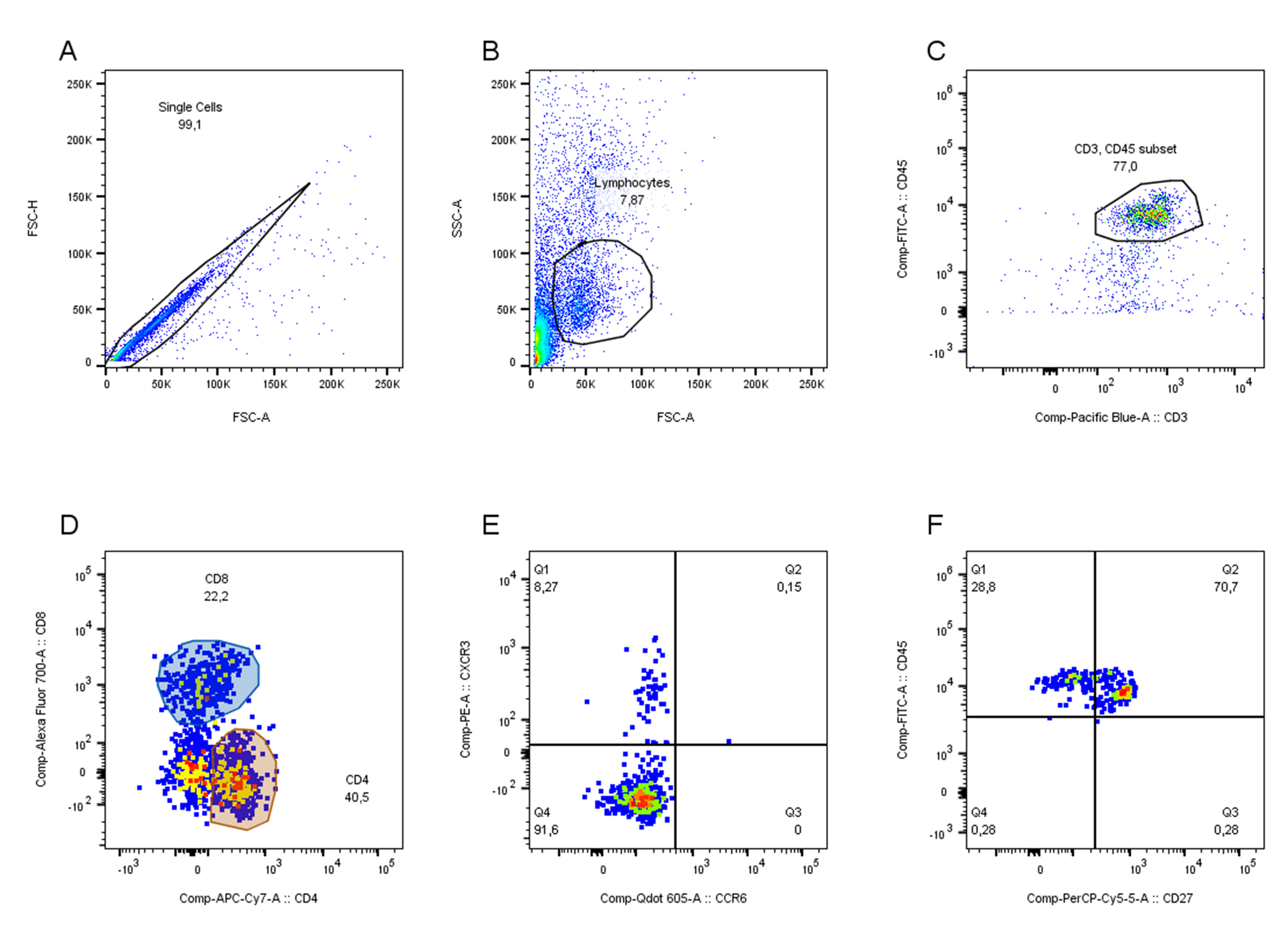

Supplement: Supplementary Figure 4 — Gating strategy display the analysis of T lymphocytes in regard to memory phenotype and function. Peripheral Blood mononuclear cells (PBMCs) were stained using the panel indicated in the method section for quantitative analysis of memory and function markers in both CD4+ and CD8+ T cells. Samples were gated on monocytes based on SSC-A (complexity) and FSC-A (size) (A), single events (B), CD3+CD45+ cells (T lymphocytes) (C), CD4 and CD8 T cells (D), CXCR3 and CCR6 (T helper subtype) (E), and CD45 and CD27 (memory markers) (F). [file Image_4.tiff]
