# Supplementary material for: Immunomodulatory and Anti-fibrotic Effects Following the Infusion of Umbilical Cord Mesenchymal Stromal Cells in a Critically Ill Patient With COVID-19 Presenting Lung Fibrosis: A Case Report
Source: Front Med (Lausanne). 2021 Nov 17;8:767291. doi: 10.3389/fmed.2021.767291 (PMC8635722; doi:10.3389/fmed.2021.767291)
Supplement: Supplementary Table 1 — List of antibodies used for the flow cytometry analyses. [file Table_1.docx]

**Supplemental Table 1: List of antibodies used for flow cytometry analyses**

| **Antibody** | **Fluorophore** | **Supplier** | **Catalog #** | **Clone** |
| --- | --- | --- | --- | --- |
| CD3 | efluor450 | eBioscience | 48-0038-42 | UCHT1 |
| NHP/CD2 | efluor 450 | eBioscience | 48-0029-42 | RPA2.10 |
| CD20 | efluor450 | eBioscience | 48-0209-42 | 2H7 |
| CD19 | efluor450 | eBioscience | 48-0199-42 | HIB19 |
| HLADR | FITC | PharMigen | 555811 | G46-6 |
| CX3CR1 | PE | eBioscience | 12-6099-42 | 2A9-1 |
| CCR2(CD192) | PerCP-Cy5.5 | BioLegend | 357204 | KO36C2 |
| CD19-5(CCR5) | APC-Cy7 | BD | 557755 | 2D7/CCR5 |
| CD14 | Per-Cy7 | BD | 557742 | M5E2 |
| CD16 | APC 700 | eBioscience | 56-0168-42 | eBioCB16(CB16) |
| CD196/CCR6 | BV-605 | BD | 562724 | 11A9 |
| TF(CD142) | APC | eBioscience | 17-1429-42 | HTF-1 |
| CD141 | PerCP-Cy5.5 | BioLegend | 344112 | M80 |
| CD11c | PE/Cy7 | BioLegend | 301608 | 3.9 |
| CD123 | PE | BD | 340545 | 9F5 |
| CD3 | efluor450 | eBioscience | 48-0038-42 | UCHT1 |
| NHP/CD2 | efluor 450 | eBioscience | 48-0029-42 | RPA2.10 |
| CD20 | efluor450 | eBioscience | 48-0209-42 | 2H7 |
| CD19 | efluor450 | eBioscience | 48-0199-42 | HIB19 |
| HLADR | FITC | PharMigen | 555811 | G46-6 |
| CD16 | APC 700 | eBioscience | 56-0168-42 | eBioCB16(CB16) |
| CD14 | Qdot605 | Lifetecnologies | Q10013 | TÜK4 |
| CD196/CCR6 | BV-605 | BD | 562724 | 11A9 |
| CD3 | efluor450 | eBioscience | 48-0038-42 | UCHT1 |
| CD8 | AF700 | eBioscience | 561026 | RPA-T8 |
| CD62L | APC | BD | 559772 | DREG-56 |
| CXCR3 | PE | PharMigen | 557185 | 1C6/CXCR3 |
| HLA-DR | PECY7 | BD | 335813 | L243 |
| CD27 | PercP-Cy5.5 | Beckman Coulter | 6607107 | 1A4CD27 |
| CD45RO | ECD | Beckman | IM2712U | UCHL1 |
| CD4 | APC-Cy7 | BD | 560158 | RPA-T4 |
| HLA-DR | PECY7 | BD | 335813 | L243 |
| CD3 | efluor450 | eBioscience | 48-0038-42 | UCHT1 |
| NHP/CD2 | efluor 450 | eBioscience | 48-0029-42 | RPA2.10 |
| CD20 | efluor450 | eBioscience | 48-0209-42 | 2H7 |
| CD19 | efluor450 | eBioscience | 48-0199-42 | HIB19 |
| CD14 | Qdot605 | Lifetecnologies | Q10013 | TÜK4 |
| CD16 | APC 700 | eBioscience | 56-0168-42 | eBioCB16(CB16) |
| IL1-β | FITC | BD | 340515 | AS10 |
| IL-2 | AF700 | BioLegend | 500320 | MQ1-17H12 |
| IFN-ɤ | PE | eBioscience | 554552 | 4S.B3 |
